# Myopic Reformers

**Published:** 1884-04

**Authors:** Geo. E. Blackham


### MYOPIC REFORMERS.

GEO. E. BLACKHAM, M. D., F. R. M. S.

Myopia is a condition in which the patient does not see distant things with any distinctness, but often sees the things which lie within his limited circle of vision with very great clearness and sharpness.

The little circle of which he is the centre and the circumference of which he can reach by stretching out his arm contains for him all that is clear and well defined in this wonderful universe, all beyond is vague, misty and unintelligible, and he comes unconsciously to neglect it entirely and to live on as though it did not exist. Myopia is usually hereditary, often progressive and always incurable; and often the patient is unaware of his infirmity, and at first but little inclined to be grateful to those who suggest that he ought to wear glasses so as to get clearer and truer views of the world at large.

The professional reformer is usually afflicted with a high degree of intellectual and moral myopia. He has discovered, perhaps, that *for him* there is no half-way house between total abstinence from the use of alcoholic liquors and inebriety, and immediately he proceeds to insists that the rule for him is the only rule for all. He sees nothing beyond his own contracted circle, and is very apt to resent, somewhat fiercely, any suggestion that intellectual "specs" would perhaps extend his mental horizon and show him that all men are not formed on a single model of which he is the perfect type.

The Civil Service Reformers seem, as a class, to be afflicted with a mental myopia of very high degree. They seem to be able to see but one thing at a time. At present this one thing is competitive examination, which is boldly put forth as the great universal panacea for all the ills our body politic is heir to.

Seeing clearly enough that government employes should have certain educational qualifications they assume that the degree of these qualifications can be determined by examination, and that the applicant passing the best examination is the best educated and therefore best fitted for the position. In fact, if our Civil Service Reformers were not so dreadfully myopic they would see that there are other and more important qualifications for positions of trust and responsibility than the ability to name the highest mountain or the longest river in the world, or to solve the problems in the fifth book of Euclid; they would see that honesty, prudence, judgment and other things not determinable by competitive examination are far more important, and that the best man for a given place is by no means sure to be first in the competition; that a man may be able to pass a very creditable examination and not be good for much else.

The diet reformers are often similarly afflicted. They can see only what affects themselves and are determined that all the world shall conform to their bill of fare. Here is a man who finds that meat does not seem to agree with him and straightway he goeth into the highways and byways proclaiming in a loud voice that the only road to health, wealth and happiness, not to mention virtue and good looks, is by way of Graham bread, wheaten grits and cold water. Another gets close to the tea kettle and presently avows his belief that it is the long-lost fountain of youth, and that to be virtuous and happy you must drink a gallon or so of hot water three times a day.

Now the curious thing about all this is that there is usually much truth in the queer ideas enunciated by these people, and that they themselves are in the main perfectly honest in their advocacy of their schemes. They tell things as they see them, and they can see certain things clearly enough, but on account of their mental or moral myopia they see only just what they specially look at, and that out of all proper proportion to its surroundings.

Caricature has been defined as "the exaggeration of a characteristic," and so the mental pictures of these mental myopes all become in a sense caricatures in which the special object which engages their attention has to be brought so close before they can see it clearly that, by the laws of perspective, it dwarfs everything else. If these good people could only be persuaded to put on philosophical spectacles to increase the range of their mental vision and then stand back a little and view the special object of their devotion from a reasonable distance, they would begin to see that their own special "fad" was not the only matter of interest and value in this great world of ours, and that civilization would not be proved a failure even if their darling reform should "die a borning."

Thus far we have considered the born mental myopes—the hopelessly incurable cases—but there is another slighter form of the trouble which is much more frequent—in fact, it is more than probable that we all suffer from it at times—it is a sort of temporary myopia, brought on by too close examination, for too long a time, of some special matter. The young mother with her first baby is a case in point—the attack is severe, but usually passes off before number two appears in this "vale of tears." In some cases each new baby brings a fresh attack. Never, never, never since the world began was there such a lovely, brilliant, marvellous baby. The mould-like fuz which covers its little scalp becomes to the mother's eye the loveliest hair, "just like papa's;" in its pink and pretty countenance are discovered all the desirable features of its ancestors for three generations past. Baby's wailings and colics, his first efforts to walk and speak become the important events in the history of the world, but after a time the young mother's mental myopia passes off and she begins to see her first born more nearly in his proper relations to the world at large, though it is doubtful if she ever is really capable of seeing him as others see him.

The man with a new patent, the doctor with a new remedy, the girl with a new lover, are all samples of the milder form of temporary mental myopia, in which the trouble is usually of brief duration and is fully and promptly recovered from.

For these mild cases it is not worth while to say anything, indeed it is scarcely desirable that anything should be done, they are sure to recover, and that promptly, if let alone and their symptoms are harmless to themselves and amusing to others. But what of the bad cases, the congenital incurables who become reformers by predestination, who can see nothing beyond the reach of their own areas and very little beyond their own noses, and who are usually morally color-blind as well as mentally myopic? What can be done for them ? What should be done for them ? Should not the State provide for them as it does for the physically lame, halt and blind, and either compel them to wear such mental spectacles as shall extend their mental vision, or shut them up where they shall be unable to torment their more normally constituted neighbors, with their absurd and distorted notions of men manners and things ?

